# Gender Differences in How Leaders Determine Succession Potential: The Role of Interpersonal Fit With Followers

**DOI:** 10.3389/fpsyg.2019.00752

**Published:** 2019-05-03

**Authors:** Floor Rink, Janka I. Stoker, Michelle K. Ryan, Niklas K. Steffens, Anne Nederveen Pieterse

**Affiliations:** ^1^ Faculty of Economics and Business, University of Groningen, Groningen, Netherlands; ^2^ Psychology, College of Life and Environmental Sciences, University of Exeter, Exeter, United Kingdom; ^3^ School of Psychology, The University of Queensland, Brisbane, QLD, Australia; ^4^ Rotterdam School of Management, Erasmus University Rotterdam, Rotterdam, Netherlands

**Keywords:** gender differences, leadership, succession, interpersonal fit, old boys network

## Abstract

This paper examined the existence of gender differences in the degree to which leaders’ perceptions of successor potential is influenced by interpersonal fit. In Study 1 (*N* = 97 leaders, *N* = 280 followers), multi-source field data revealed that for male leaders, ratings of followers’ potential as successors were positively related to interpersonal fit, measured by the degree to which followers’ saw their leadership as being close and interpersonal (i.e., being coaching, transformational, and leading by example). For female leaders, these relationships were absent, suggesting that they are less influenced by interpersonal fit. In Study 2 (*N* = 311 leaders), a scenario study provided causal evidence that male leaders rated potential successors more positively when they perceived greater interpersonal fit with followers, whereas female leaders’ successor ratings were not informed by perceptions of fit. We discuss the theoretical and practical implications for gendered leadership successor perceptions in organizations.

## Introduction

The relatively slow pace at which women’s careers develop is a timely research topic in the fields of psychology and management. Scholars aim to understand why women still face obstacles in being promoted into senior leadership positions (Vincent-Lancrin, 2008), even though they are currently more successful than men in earning advanced educational degrees and are increasingly participating in the labor market. For example, the last global McKinsey report on women in the workplace (McKinsey and Company, 2017) demonstrates that while women are not leaving their companies at higher rates than men do, they still only make up, on average, only 20% of our senior corporate leaders.

The vast amount of research on the underrepresentation of women in positions of leadership suggests that disparities in promotion rates is not caused by women’s lack of desire to advance their career (e.g., Ellemers et al., 2012; Peters et al., 2013). Rather, compared to men, women are less optimistic about their opportunity to attain a leadership position and anticipate more difficulties once in such positions, which makes them doubt their leadership competencies (Keller et al., 2013, see also Lockwood and Kunda, 1997). Indeed, research has repeatedly validated that biased treatments during leader selection processes significantly contribute to women’s disadvantage (Burke, 2011).

Although scholars offer various explanations for the existence of gendered selection biases, two reasons stand out. First, internalized gendered beliefs (or stereotypes) about what it takes to be an effective leader have been shown to lead to gender bias (Eagly et al., 1992; Heilman, 2001; Eagly and Karau, 2002). Specifically, both men and women tend to endorse the belief that effective leaders should show stereotypically masculine or agentic traits (i.e., the think manager-think male association, Schein, 1975, see also Davison and Burke, 2000).

Second, it has been argued that when it comes to leadership succession decisions, those at the top, who are overwhelmingly men, show a preference for promoting others with whom they share similar traits and characteristics or those with whom they have a positive interpersonal relationship. A recent meta-analysis (Koch et al., 2015) suggests that both stereotypes and interpersonal liking or similarity mutually reinforce each other. In this way, a preference for leadership successors that match traditionally (male) notions of leadership also enhances similarity at the top of organizations. This process, in turn, further limits the career possibilities of those who do not fit within a masculine culture.

Notably, while there is robust support for the existence for internalized stereotypes about leadership and gender and the way in which they bias selection and succession decisions (e.g., Heilman, 2001; Eagly, 2007; Cuddy et al., 2015), research investigating whether male leaders preference for socially similar others in leadership positions has been inconclusive. Literature from the fields of economics and sociology suggest that male leaders are motivated to maintain elite informal networks structures on the basis of interpersonal fit (i.e., the so-called “old boys network”, McDonald, 2011). Interpersonal fit refers to the existence of positive interpersonal relationships guided by social similarities that facilitate the exchange of information and resources among those involved (e.g., Byrne, 1971; Ibarra et al., 2005). However, it remains unclear whether women, once they are in leadership positions, also make succession decisions on the basis of interpersonal liking due to experiences of social similarities or shared important features. Given that women are progressing into the upper organizational echelons (although there is a long way to go before equal representation is reached), it becomes more prudent and possible to answer this question. If women do make promotion and succession decisions based on interpersonal fit, this would suggest a breaking down of the old boys’ network and a facilitation of the number of women in leadership positions over time. In this paper, we therefore systematically examine the degree to which male and female leaders rely on social similarities and use interpersonal fit when informally selecting successors for a future leader role.

## Study Overview

We will use two distinct research methods to examine our research question. To ensure external validity and yield generalizable results, we first conducted a study among leaders and their followers in the field (*N* = 97 leaders, 24% female and *N* = 280 followers). Here, we asked leaders to indicate how much they would endorse each of their followers as a successor for a leadership position. To prevent common method bias among the study variables (Podsakoff et al., 2003), interpersonal fit was approximated by asking followers to rate their leader on three relational leadership styles: coaching, transformational leadership, and leading by example (Bass and Avolio, 1990; Arnold et al., 2000). Coaching entails helping followers to advance in their career and provide guidance in improving their skills; leading by example involves leader actions that influence followers to behave in ways that they consider valuable (Arnold et al., 2000); and transformational leadership refers to leaders who are visionary and inspire followers to perform beyond leader expectations (e.g., Bass and Avolio, 1990). Although positive follower ratings of these three leadership styles do not capture interpersonal fit directly, such ratings are known to be associated with positive relationships and a sense of sharedness (Wood, 2000; Wang et al., 2018). This is in line with Byrne’s (1971) similarity-attraction hypothesis that positive relational assessments rarely occur without an underlying source of social similarity. Hence, positive leader evaluations on these three dimensions likely represent a good proxy of interpersonal fit. However, to further test the robustness of our Study 1 findings, we also conducted a second experimental scenario study among leaders (*N* = 311 leaders, 44% female). In this study, we established the isolated and causal effects between leader gender, interpersonal fit, and leader ratings of followers’ successors potential. Participants were asked to imagine themselves in a position where they had to evaluate leadership succession candidates in the presence or absence of interpersonal fit information. Before turning to these studies, we will first review the literature and develop our hypothesis in more detail.

## Theoretical Framework

### Male Leaders and Interpersonal Fit

One of the main explanations for the underrepresentation of women in leadership positions is the “think manager-think male” association (Schein and Davidson, 1993; Sczesny, 2003), leading to masculine norms for career progression (Crosby et al., 2004). This association reflects the robust belief that men are more prototypical as leaders and enjoy higher status in society than women do (Eagly, 1987, 2007; Davison and Burke, 2000; Heilman, 2001). Although views of effective leadership have gradually shifted over time to become somewhat congruent with more stereotypically feminine traits, such as warmth, good communication, and strong people skills (Bass et al., 1996; Eagly and Johannesen-Schmidt, 2001; Koenig et al., 2011), studies repeatedly confirm that both men and women tend to describe effective leaders as possessing mostly masculine traits (Koenig et al., 2011).

However, Koch et al. (2015) found meta-analytical evidence that men’s preference to hire other men in male-dominated jobs was relatively stronger than was women’s preference. As a possible explanation for this finding, the scholars argued that, in addition to holding gendered beliefs about leadership, male leaders also feel more socially similar to, or experience more interpersonal fit with, prospective male successors, which leads them to evaluate these successors more positively (see also Byrne, 1971). The general metaphor used to illustrate this male preference for interpersonal fit is the so-called “old boys’ network.” This metaphor captures what Kanter (1977) coined as the “shadow structure” within organizations, whereby male leaders tend to engage with those junior employees, or followers, within their informal network activities who are also male or who are socially similar to them. The theoretical rationale currently used to explain why male leaders hold this preference is that they expect these followers to work in similar ways and believe that these followers will endorse their leadership style, which further legitimizes current power relations (Ibarra, 1992; McDonald, 2011). However, this “old boys’ network” thereby often excludes women and members of other minority groups, such as those based on race, religion, class, or sexuality.

The old boys’ network argument thus represents a second potential explanation for men’s continued domination in leadership positions. Organizational and sociological studies have consistently established the clear benefits of having a strong informal management network for followers, as networks play a key role in identifying and preparing potential successors for future leadership roles (Garman and Glawe, 2004; Virick and Greer, 2012). For example, according to the sponsored mobility model of career success, informal networks give (male) followers access to the valuable resources and support needed to stand out and advance in their careers (McDonald, 2011). In this way, men not only experience greater role congruity in senior positions than do women, they are also more likely to receive more social support on their route to the top (Saloner, 1985; Simon and Warner, 1992; Oakley, 2000; Forret and Dougherty, 2004; Bu and Roy, 2005; Hogan et al., 2005; Berardi and Seabright, 2011; Kramarz and Thesmar, 2013).

While it is clear that male leaders’ preferences for interpersonal fit with their followers and potential successors would further reinforce gender inequality dynamics, direct evidence for the existence of this preference is relatively scarce (McDonald, 2011; Renneboog and Zhao, 2011). However, one meta-analysis across 30 studies in economics (Ng et al., 2005) demonstrates that most organizations have sponsor systems in place, in terms of career sponsorship from senior managers, formal supervisory support, developmental opportunities, and access to organizational resources. These sponsor systems, in turn, determine the upward mobility (salary increases and the number of career steps made) of followers who are closely connected to higher management (i.e., those who are also male, married, and white, Ng et al., 2005).

From a psychological view, there has been, to our knowledge, very little direct evidence for the old boys’ network, although the idea does resonate with similarity attraction theory, to which we referred earlier (Byrne, 1971). This theory emphasizes the importance of interpersonal processes between individuals, such as between leaders and their potential successors. Corresponding to the concept of homophily, it posits that in starting new relationships, individuals have the tendency to associate with, and develop a greater liking for others based on shared characteristics (e.g., Greenberg and Mollick, 2017) and/or shared social attitudes (i.e., similar beliefs toward a certain idea, person, or situation, Berscheid and Walster, 1969; Eagly and Chaiken, 1998). Psychological studies offer parsimonious support for this assumption, showing that people hold a preference for shared attitudes because it leads them to expect that one’s own beliefs are correct, the other will demonstrate predictive behavior, and the other will like them and offer support if needed (e.g., Wood, 2000).

Hence, scholars within this field also argue that within traditional male-dominated organizations, male leaders should be inclined to select potential successors for managerial positions with whom they share social similarities (McCarthy et al., 2010). A recent network study among scientists suggests that this pattern may indeed exist, showing that men, compared to women, build professional networks with a higher proportion of male to female supporters (both inside and outside their academic institution), and this proportion, subsequently, relates to higher scores of men on perceived career success and mobility (Spurk et al., 2015). In conclusion, research from different disciplines suggests that male leaders will show a tendency to informally select potential successors based on interpersonal fit perceptions.

### Female Leaders and Interpersonal Fit

An important next question is whether we would expect female leaders, just like male leaders, to be attuned to interpersonal fit perceptions when selecting successors. Literature on the old boys’ network phenomenon and similarity attraction processes proposes two competing perspectives on the way in which female leaders, once in power, influence the career prospects of their followers (Maume, 2011; Stainback et al., 2016).

On the one hand, scholars argue that female leaders are indeed susceptible to the laws of homophily and similarity attraction, implying that they too will be inclined to take interpersonal fit into account when choosing potential successors for management positions. In this way, scholars have argued that female leaders can act as “change agents,” and that their presence in leadership positions will automatically erode gender inequality in the work place (McPherson et al., 2001; Elliott and Smith, 2004). Indeed, there is evidence that female leaders are just as likely as men to provide networking opportunities to followers who are socially similar to them (e.g., Konrad et al., 2008). Building on this logic, Greenberg and Mollick (2017) go one step further and coined the term “activist choice” to argue that interpersonal fit will probably be *more* salient to female leaders than to male leaders because of “…perceptions of shared structural barriers stemming from a common group-level social identity and an underlying desire to help overcome them” (p. 342).

On the other hand, scholars have underscored a “cog in the machine” perspective on female leaders’ attitudes and behaviors toward their followers (Cohen and Huffman, 2007). This perspective argues that there are a number of reasons why female leaders may *not* be in a position to change existing gender inequality. First, although the study of Koch et al. (2015) showed that male leaders preferred men to women in male-dominated jobs more strongly than female leaders did, female leaders still generally held a male preference. This finding underscores the large body of psychological research demonstrating that women too tend to endorse the “think male-think manager association” (Eagly, 2007). Hence, women seem, at least under some circumstances, also biased in which followers they view as possible future leaders (Powell et al., 2002). Second, it is also likely that in their leadership roles, women do not hold sufficient power to challenge the old boys’ club, and hence cannot simply create a “new girls’ network” (as awful as that term is). This lack of power is due to the facts that women simply tend to occupy lower level positions compared to their male counterparts (Elliott and Smith, 2004) and are often taken minorities in they do obtain top-level positions (Ellemers et al., 2012). Finally, if women must display stereotypically masculine traits to be seen as suitable for leadership positions (Heilman, 2001, 2012), women may accommodate to these expectations and will start to value these features as positive traits (Maume, 2011). Consequently, female leaders can also conform to existing management norms and behave like male leaders, hereby further advancing the careers of male followers, rather than that of female followers (Kanter, 1977; Ely, 1994). For example, scholars have introduced the “queen bee phenomenon”, suggesting that in male-dominated organizations, female leaders can start distancing themselves from junior women psychologically and begin to legitimize gender inequality as a coping response to their marginalized position (Derks et al., 2016). In support of this idea, studies have found that women who are advancing in their career adopt a masculine self-presentation when they experience social identity threat due to gender biases (Ellemers et al., 2012; Kaiser and Spalding, 2015).

In relation to interpersonal fit perceptions more directly, the literature seems to offer support for both perspectives. Psychological research has found that, generally speaking, both interpersonal fit perceptions and gender significantly determine both male and female leaders’ interpersonal attraction to followers (Tsui and O’Reilly, 1989). Moreover, research has found support for the “activist choice” notion (Greenberg and Mollick, 2017), particularly when women’s representation at higher organizational levels is substantial (Cohen et al., 1998, also see Stainback et al., 2016) or when female leaders hold positions for longer periods of time (Arvate et al., 2018). These findings suggest that female leaders too can be guided by interpersonal fit preferences and hereby shape the career positions of other women. Nonetheless, in contrast to these findings, there is also research clearly supporting the opposing perspective, showing that female leaders’ direct impact on the careers of female followers is limited (Maume, 2011; Stainback and Kwon, 2012).

It is also important to note that the extant literature does not, to our knowledge, systematically compare the relevance of interpersonal fit between female and male leaders. It is therefore useful to build on sociological and management studies that have looked more closely at the role of social similarity and fit in the network building activities of male and female leaders (e.g., Ibarra, 1992; Benenson, 1993; Baumeister and Sommer, 1997; Friebel and Seabright, 2011). In this area of research, studies obtained clear gender differences in network relationships (Ibarra, 1992; Lyness and Thompson, 2000; Metz and Tharenou, 2001; Linehan and Scullion, 2008; Kleinbaum et al., 2013). In Ibarra’s seminal work (Ibarra, 1992, 1993), for example, men were more likely to form homogeneous ties across multiple networks based on same sex and fit than women. In addition, men’s ties were also significantly stronger than women’s ties. In line with the “cog in the machine” perspective, these findings strongly support the idea that for female leaders, similarity to potential successors may be less relevant than for male leaders.

Based on the network findings above, which most closely reflect leader responses to social similarity, our central proposition is that the relative importance of interpersonal fit when evaluating followers for their potential as successors is likely to be gendered. More specifically, we hypothesize that leader gender will influence the link between interpersonal fit and leader ratings of followers’ successor potential, such that male leaders will be more likely to select interpersonally similar followers as potential successors than will female leaders. In the following sections, we will present two studies, a multisource field study and a vignette study among male and female leaders, in which we examined our proposition.

## Study 1

In our first study, we conducted a field survey where we examined both leaders and their followers as research sources. In this way, we could test the degree to which interpersonal fit perceptions affected male and female leaders’ evaluations of their followers’ potential as successors. As mentioned in section “Introduction,” we captured interpersonal fit by asking the followers to rate their leaders on coaching, leading by example, and transformational leadership, as these three relational leadership styles generally reflect positive relationships and social similarity between leaders and their followers (Judge and Piccolo, 2004; Barbuto and Burbach, 2006). Coaching reflects adequate guidance in career advancement and skill development; leading by example reflects leader actions that followers value and like to adapt (Arnold et al., 2000); and transformational leadership reflects the use of ideals that inspire followers (e.g., Bass and Avolio, 1990). Notably, scholars rely heavily on follower ratings to assess leader evaluations because leadership inherently entails the dynamic interaction between leaders and followers (Riggio et al., 2008). Moreover, by measuring interpersonal fit unobtrusively through independent follower ratings, we were able to circumvent common method bias (such as inflated relationships between perceptions of interpersonal fit and leaders’ successor ratings; Siemsen et al., 2010). Finally, scholars studying the “old boys’ network” in organizations or similarity-attraction processes between leaders and followers generally expect a close link between positive follower ratings of leaders and leader-follower similarity. This is evident from the notion that socially similar followers will endorse their leaders (McDonald, 2011). Moreover, Byrne’s (1971) interpersonal fit definition highlights that fit consists of social similarity, positive interpersonal relationships, and information exchange because “likeness begets liking.” From this argument, it follows that positive leader evaluations by followers indirectly capture underlying fit perceptions. Given our focus on these three leadership styles, our formal first hypothesis is:

H1: Leader gender will moderate the positive relationships between interpersonal fit (i.e., follower evaluations of leaders’ coaching, leading by example, and being transformational) and leaders’ ratings of follower’s potential as successor. This relationship should be significantly stronger for male leaders than for female leaders.

### Method

#### Participants

Our participants were 290 followers and 97 leaders. As 10 followers did not indicate their demographic data, our final sample consisted of 280 followers. Participants were employed in six Dutch Ministries and were invited to participate *via* internal email. The overall respective department tenure from all participants ranged from less than 1–43 years (*M* = 14.50; *SD* = 11.03).

Of the followers, 102 were female and 179 male, with a mean age of 47 years. Followers themselves held mid-level management positions and were highly educated, with 97% of them having completed a university degree. On average they had worked for approximately 14 years in their ministry (*SD* = 10.79) and 3 years in their current role (*SD* = 3.35). Of the leaders, 23 were female and 74 male, their mean age was 49 years and 93% held a university degree. On average, they worked for approximately 16 years in their ministry (*SD* = 12.43) and also 3 years in their current role (*SD* = 5.42).

#### Procedure

Participation was voluntary, and confidentiality was assured. All measures were translated into Dutch using a double-blind back-translation procedure. To rule out any common method bias in responses, leaders evaluated their followers for their potential as a leadership successor, whereas the followers rated interpersonal fit in terms of the extent to which their respective leader coached them, led them by example, and used a transformational leadership style.

#### Measures

Leaders indicated a *follower’s potential to be a leadership successor* by responding to one item on a seven-point Likert scale ranging from 1 (*strongly disagree*) to 7 (*strongly agree*); “Would you support and endorse this person as the leader of the group after you had gotten a promotion and needed a replacement?” *Leader gender* and *follower gender* (as a control) were coded as 0 = male, 1 = female.

To assess interpersonal fit, we measure followers evaluations of their leaders on the following: first, leaders use of *coachin*g, where followers responded to the following items on identical seven-point Likert scales (Arnold et al., 2000; *α* = 0.88); “My leader helps us our team in areas in which we need more training”; “My leader encourages team members to solve problems together”, “My leader helps developing good relations among team members”. Second, *leadership by example* (Arnold et al., 2000; *α* = 0.86), where followers answered two items: “My leader sets high standards for performance by his/her own behavior,” “My leader sets a good example by the way he/she behaves”. Finally, we used six items to measure followers’ perceptions of how *transformational* their leader was (Carless et al., 2000, *α* = 0.85); “My leader inspires others with his/her plans for the future”, “My leader leads by example,” “My leader develops a team attitude and spirit among employees,” “My leader insists on only the best performance,” “My leader shows respect for my personal feelings,” “My leader has stimulated me to rethink the way I do things.”

### Results

Table 1 presents the descriptive statistics. Our proxies for interpersonal fit were all significantly and positively related to one another (coaching and leading by example, *r* = 0.81, *p* < 0.001; coaching and transformational leadership, *r* = 0.75, *p* < 0.001; leading by example and transformational leadership, *r* = 0.86, *p* < 0.001). There were also significant relationships between follower gender and successorship (*r* = 0.15, *p* < 0.05) as well as perceived leader coaching (*r* = −0.11, *p* < 0.05), suggesting that this variable should be included in our hypothesis testing analyses (Becker, 2005).

**Table 1:** Descriptive statistics and Pearson zero-order correlations (study 1).

Variables	*M*	*SD*	1	2	3	4	5	6
1. Coaching	4.95	1.25	–	0.81^**^	0.75^**^	0.08	0.05	−0.11^*^
2. Leading by example	5.10	1.03		–	0.86^**^	0.05	0.06	−0.02
3. Transformational leadership	5.23	1.33			–	0.06	–0.00	−0.09
4. Leader gender	1.22	0.41				–	−0.01	0.11
5. Successorship	3.89	2.01					–	0.15^*^
6. Follower gender	1.34	0.47						–

Note: All correlations are at the individual level of analysis. Correlations involving these variables should therefore be interpreted with caution. n = 280,

* p < 0.05,

** p < 0.01. Two-tailed.

Given that the number of followers per leader was somewhat low (*M* = 3.07; with 16% of leaders having a single follower), multi-level analyses would likely yield inaccurate estimates (the number of observations are lower than a minimum of 15–20 that researchers’ simulations have shown to be optimal; Hox, 2010). We therefore focused on single-level analyses and ran a series of hierarchical linear regressions through Hayes’ PROCESS macro (Hayes, 2013, Model 1, 95,000 bootstraps, 95% *CI* levels). For this purpose, we standardized all model variables, except for leader gender and follower gender (Bickel, 2007). The regression models we ran assessed whether the interpersonal fit between followers’ leader evaluations (i.e., in terms of evaluations of leaders’ coaching, leading by example, and being transformational) and leader evaluations of followers’ successorship potential was moderated by leader gender.

Generally speaking, the results supported Hypothesis 1. There were no significant main effects for leader gender (lowest *p* = 0.62). The three proxies for interpersonal fit each had a significant direct relationship with followers’ successorship potential (coaching, *p* < 0.001; leading by example, *p* = 0.014, and transformational leadership, *p* = 0.004). As anticipated, however, these main effects for the proxies were qualified by significant interaction effects with leader gender (i.e., coaching × leader gender, *p* < 0.001, *CI* −1.11 to −0.27; leading by example × leader gender, *p* = 0.014, *CI* −0.98 to −0.11; transformational leadership × leadership, *p* = 0.009, *CI* –1.29 to −0.18). Notably, follower gender was a significant predictor of follower successorship (lowest *p* = 0.004), but this variable did not influence our obtained findings or separately interact with any of our study variables.

**Figure 1:**
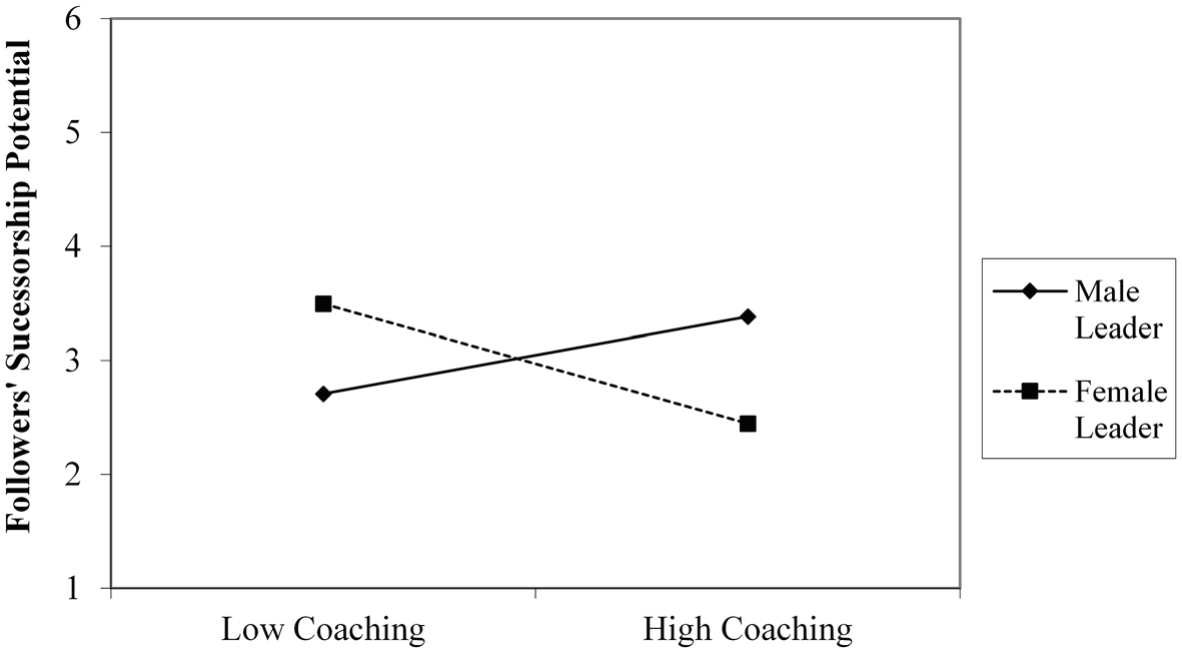
Interactive relationship of followers’ evaluation of leaders’ use of coaching and leader gender with leaders’ assessment of followers’ successorship potential (study 1).

**Figure 2:**
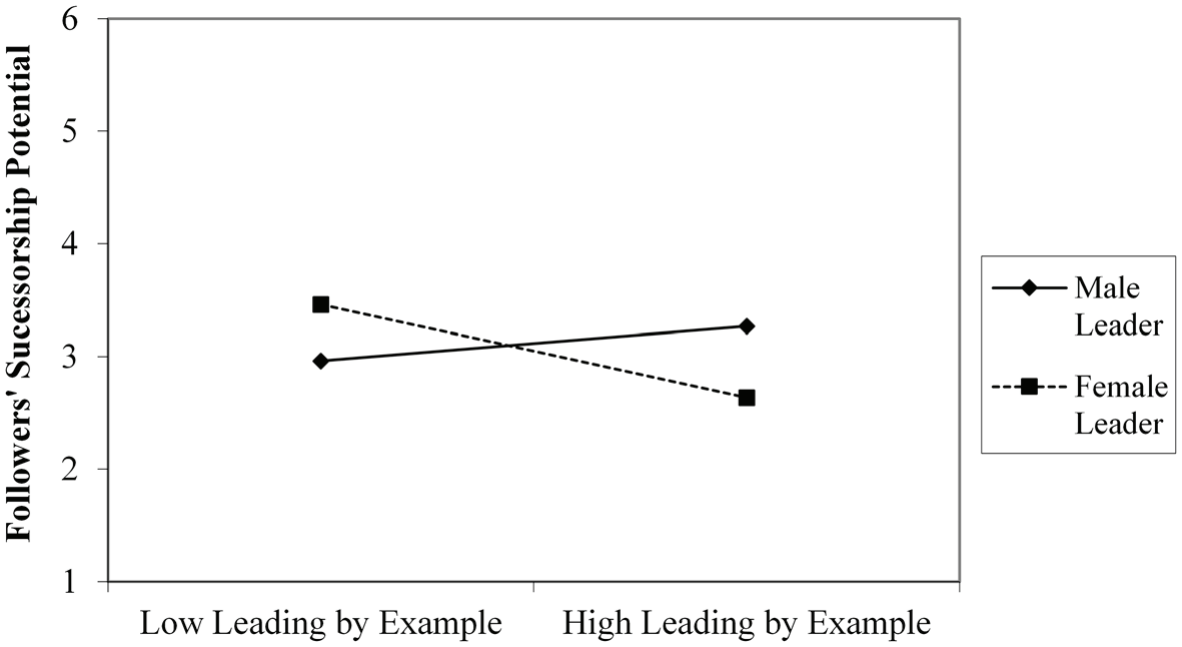
Interactive relationship of followers’ evaluation of leaders use of leading by example and leader gender with leaders’ assessment of followers’ successorship potential (study 1).

**Figure 3:**
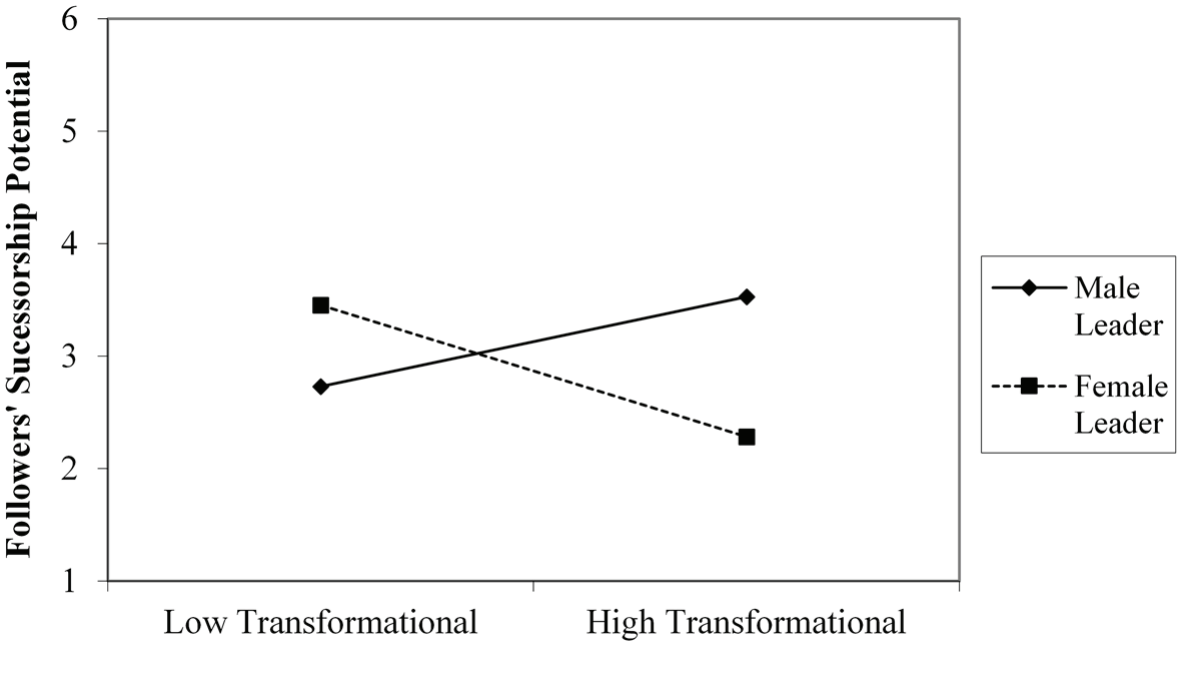
Interactive relationship of followers’ evaluation of leaders use of a transformational style and leader gender with leaders’ assessment of followers’ successorship potential (study 1).

Further decomposition of the interaction terms with simple slope analyses demonstrated that for male leaders, interpersonal fit, in terms of follower evaluations of leaders’ coaching and use of a transformational style, was significantly related to their perceptions of followers’ successorship potential (respectively, *B* = 0.17, *t* = 2.67, *p* = 0.008, *CI*: 0.045–0.30; *B* = 0.15, *t* = 2.40, *p* = 0.02, *CI*: 0.023–0.28). Only evaluations of leading by example was unrelated to perceptions of followers’ successor potential, *B* = 0.08, *t* = 1.48, *p* = 0.14, *CI*: −0.03 to 0.23). By contrast, for female leaders, interpersonal fit, in terms of follower evaluations of these leaders’ coaching (*B* = −0.26, *t* = −2.23, *p* = 0.03, *CI*: −0.49 to −0.03) and leading by example (*B* = −0.26, *t* = −2.01, *p* = 0.04, *CI*: −0.52 to −0.01) were unrelated to their perceptions of followers’ successorship potential, while their use of a transformational style (*B* = −0.23, *t* = −1.73, *p* = 0.09, *CI*: −0.048 to 0. 03) was negatively related to their successorship judgments (Figures 1–3).

#### Supplementary Analysis

The three leadership styles together also formed a highly reliable scale (*α* = 0.93). Additional regression analyses also demonstrated a similar pattern of results. There were main effects of the overall leader evaluation measure (*p* = 0.002) and follower gender (*p* = 0.005) on leaders’ ratings of followers’ successor potential. Yet again, these was also a significant interaction between leader evaluations and leader gender on their successorship ratings (*p* = 0.003, *CI*: −1.29 to −0.27). The simple slope analyses revealed that the general leader evaluations were significantly positively related to male leaders’ perceptions of followers’ successor potential (*B* = 0.15, *t* = 2.38, *p* = 0.02, *CI*: 0.03–0.28) but significantly negatively related to female leaders’ successor ratings (*B* = −0.26, *t* = −2.12, *p* = 0.03, *CI*: −0.51 to −0.09). See our Supplementary Figure 1.

#### Discussion

Study 1 represents a unique field sample of senior leaders and their followers. To rule out common-source bias (Podsakoff et al., 2003), this study used multiple sources such that senior leaders provided data concerning leadership successor potential of followers, while followers provided a proxy for interpersonal fit through their evaluations of their leaders. Notably, we employed a continuous measure of successor potential that allowed for assessing natural variation in potential and its relationship with interpersonal fit. Hence, this study provided externally validating evidence of our hypothesized relationships.

Nonetheless, Study 1 is not without limitations. First, our reliance on follower ratings of the three relational leadership styles implies that we did not capture interpersonal fit directly. Hence, it could be that followers’ evaluations merely reflect positive leadership behaviors, rather than an underlying source of similarity. Moreover, given that followers can make incorrect inferences of leadership behavior (see e.g., Bono et al., 2012), it would have been more optimal if leaders would have also provided self-ratings on the leadership styles. Interestingly, although the recent meta-analysis conducted by Wang et al. (2018) shows that leader-follower ratings of the three specific leadership dimensions, we focus on generally correlate significantly with each other, suggesting that our follower ratings probably reasonably reflect leaders’ own perceptions. Even so, we recognize that the use of multiple data sources to assess similar constructs is important because it circumvents biased response patterns. Moreover, relevant in our case, it would have also allowed us to develop and use a more direct indicator of fit.

Second, given the cross-sectional nature of this study, another alternative explanation of our findings may be that followers themselves, once chosen as a successor by a male leader, start to evaluate leadership and interpersonal fit more positively, whereas for female leaders, the reverse may be true. Such an interpretation would suggest that followers’ evaluations of their female leaders are not influenced by the extent to which these leaders identify them as a successor. This alternative logic seems unlikely as it goes against recent research demonstrating that successors generally positively evaluate their work environment and their leader (Van Quaquebeke et al., 2011; Steffens et al., 2018). Yet we conducted a second experimental vignette study to more directly test whether interpersonal fit perceptions have a greater role in successor ratings of male leaders than that of female leaders and to establish the causality of this claim. We thus designed Study 2 to examine the internal validity and robustness of our hypothesis. We presented male and female leaders with three different successor profiles that varied systematically in terms of interpersonal fit. This time, we operationalized interpersonal fit between leaders and their followers in terms of commonalities that are relevant in the work domain (i.e., having the same interpersonal leadership style, using similar problem solving or absence of information on interpersonal fit with leaders). One additional profile presented a neutral baseline condition, in which no information about the presence or absence of interpersonal fit was provided. Our key dependent measure was again leaders’ ratings of follower successor potential. As this design differs from the first study, the hypothesis we tested here was:

H2: Leader gender moderates the relationship between interpersonal fit and followers’ successorship potential, such that this relationship is significantly stronger for male leaders than for female leaders.

## Study 2

### Method

#### Design and Participants

Study 2 received ethical approval by the first author’s academic institution and consisted of a leader gender (male vs. female) × interpersonal fit (control vs. fit vs. lack of fit) experimental design. Participants were 329 employees from the Dutch healthcare and financial industries. Participants all had leadership positions within their organization and participated in the study as part of an executive leadership training program. Participants were randomly assigned to one of the three interpersonal fit conditions. Thirteen participants indicated that they did not yet occupy a leadership position and five participants did not fully complete the questionnaire, which led to a final sample of *n* = 311 participants (44% female and 56% male) whom we included in the analysis. Participants’ mean age was *M* = 44.68 years, *SD* = 10.59 and their average work experience in the current position was *M* = 6.10 years; *SD* = 6.84.

#### Procedure

Participation was voluntary and confidentiality was assured. Participants were first asked to provide demographic background information and to answer several questions about their position within their organization (see below). Leaders were then asked to immerse themselves in the situation presented to them. Specifically, they had to imagine that they had been promoted at work in a more senior leadership position. As a result of this promotion, their current leadership position would become vacant. Participants were then told that one of their followers had expressed interest in their current position. They were asked to provide higher management with information about this followers’ successor potential.

There were three conditions: a follower without fit information or with information in which the follower either clearly *had* or clearly *lacked* interpersonal fit with the leader. Notably, across the three conditions, we kept the competence level of the potential successor equally high such that all leaders were presented with the same baseline information about their followers’ work performance. This procedure allowed us to compare male and female leaders’ responses to interpersonal fit while holding their expectations of the followers’ performance constant.

#### Successor Fit Manipulation

Participants in all conditions read the following description on the follower: “The candidate has the recommended diplomas, followed several management training courses, and has gained leadership experience. The candidate has been working within the organization for several years now. You are familiar with this person and believe that they will fit well in your team. Thus, this person seems competent to perform your job the candidate and can become a core member of the team.”

When participants were allocated to the control condition, they received no additional information about interpersonal fit. However, when participants were allocated to one of the two fit conditions, they did receive additional interpersonal fit information, indicating whether or not the leader had an interpersonal fit with the successor (respectively fit vs. lack of fit); “*In addition* (*However*), you feel that this person is quite *similar* (*different*) to you. The candidate has *the same* (*a different*) interpersonal leadership style, approaches problems *using a similar* (*from a different*) perspective, and holds a *similar* (*different*) work attitude.”

### Manipulation Checks and Dependent Measures

#### Measures

To check whether our manipulation of interpersonal fit was successful, we measured *perceptions of interpersonal fit* with the follower through the following three items, each rated on a seven-point Likert scale ranging from 1 (*strongly disagree*) to 7 (*strongly agree*); “I personally like the candidate,” “I think that I could personally get along with this candidate,” “I expect that it would be easy to develop a bond with this candidate,” and “I personally feel connected with the candidate” (Peters et al., 2015, *α* = 0.90).

Our central outcome variable, *followers’ successor potential*, was obtained with the following five leader statements adapted from Ryan and Haslam (2005); “I think that the candidate will be suitable for my position,” “I think the candidate will be effective in my position,” “I think the candidate will perform well in my position,” and “I think this person is an attractive candidate for the position.” These items were also assessed on a scale ranging from (1) *strongly disagree* to (7) *strongly agree* (*α* = 0.92).

As control variables, we asked leaders to estimate *the percentage of female leaders* currently working in their organization, as this signals how socially isolated these women are in their position. In turn, this unique experience could influence the succession planning of female leaders. Leaders could choose one of three categories: (1) 0–20% (46% response), (2) 21–50% (35%), or (3) >50%. The percentages of leader responses within each of these categories were, respectively, 46, 35, and 17%. Finally, for similar reasons, we also assessed leaders own *power* levels within the organization, with three items from Maner and Mead (2010); “In this position, I am able to influence others,” “I am successful in reaching my goals,” and “I control important resources of the organization.” The answer-scale was again ranging from (1) *strongly disagree* to (7) *strongly agree* (*α* = 0.72).

### Results

Table 2 presents the descriptive statistics. As anticipated, the three interpersonal fit conditions were significantly related to our dependent variables (i.e., interpersonal fit, *r* = −0.21, *p* < 0.05 and successorship, *r* = −0.25, *p* < 0.05), and these variables were also related to each other (*r* = 0.50, *p* < 0.05). These correlations indicate that fit and successorship associated negatively with our lack of fit condition (which was labeled with the highest score: 1 = presence of fit, 2 = control, and 3 = lack of fit). Of the control variables, the average percentage of female leaders currently working within an organization was significantly related to leader gender, (*r* = −0.16, *p* < 0.05), meaning that the female leaders often worked in organizations with few other women in leadership positions. However, this measure did not significantly relate to our study variables and was therefore excluded as a control to prevent biased parameter estimates (Becker, 2005). Leader power did significantly relate to interpersonal fit (*r* = 0.13, *p* < 0.01) and successorship (*r* = 0.22, *p* < 0.05), suggesting that this variable warrants special attention in our hypothesis testing. An additional one-way ANOVA with leader gender as predictor on leaders’ social power confirmed that male leaders reported having significantly more power (*M* = 5.36, *SD* = 0.95) than did female leaders [*M* = 5.06, *SD* = 0.92; *F*(1, 310) = 6.43, *p* = 0.012, *η* = 0.21].

**Table 2:** Descriptive statistics and Pearson zero-order correlations (study 2).

Variables	*M*	*SD*	1	2	3	4	5	6
1. Leader gender	1.45	0.49	–	−.05	0.20^**^	−0.16^**^	0.03	0.10
2. Fit conditions	1.93	0.83		–	0.02	−0.04	−0.21^**^	−0.25^**^
3. female leaders	1.70	0.74			–	−0.07	−0.08	0.00
4. Leader power	5.23	0.95				–	0.13^*^	0.22^**^
5. Interpersonal fit	4.89	0.96					–	0.50^**^
6. Successorship	5.29	0.88						–

Note: All correlations are at the individual level of analysis. n = 280,

* p < 0.05,

** p < 0.01. Two-tailed.

To check whether our interpersonal fit manipulation was successful, we first performed a 2 (leader gender) by 3 (interpersonal fit) ANOVA on our fit check. As intended, the results revealed a main effect of fit across the three experimental conditions that was not influenced by leader gender, *F*(2, 309) = 18.68, *p* < 0.001, *η* = 0.11. On average, all leaders rated relatively high levels of fit but reported stronger interpersonal fit with the potential successor in the fit condition, *M* = 5.27, *SD* = 0.89, than in the lack of fit condition, *M* = 4.45, *SD* = 0.96, or in the control condition, *M* = 4.96, *SD* = 0.84.

**Figure 4:**
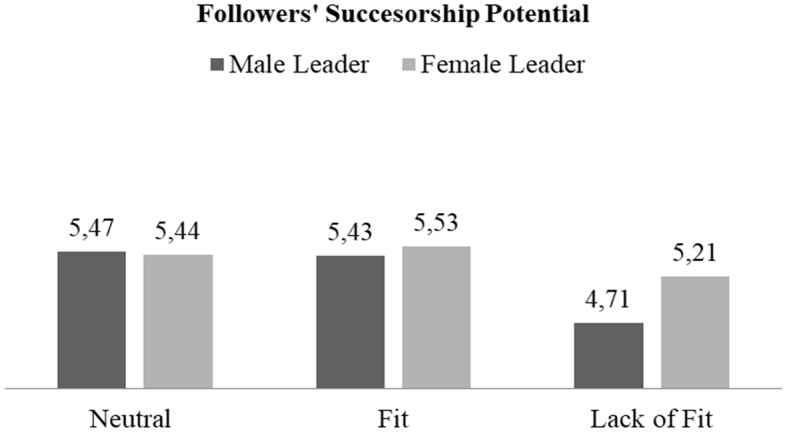
Interactive relationship of follower fit and leader gender with leaders’ assessment of followers’ successorship potential (study 2).

Our second hypothesis postulates that the relationship between interpersonal fit and followers’ successorship potential should be stronger for male leaders than for female leaders. To test this hypothesis, we ran a *second* 2 (leader gender) by 3 (interpersonal fit) ANOVA on our fit check our main outcome variable, *followers’ successor potential.* The results of this ANOVA showed that there were main effects for leader gender [*F*(1, 309) = 4.11, *p* = 0.014, *η* = 0.01], and interpersonal fit [*F*(1, 309) = 12.13, *p* < 0.001, *η* = 0.07]. However, we also obtained a marginally significant interaction effect between these two factors, *F*(2, 308) = 2.66, *p* = 0.07, *η* = 0.02. Planned comparison analyses across conditions (Tukey LSD; using 95% bootstrap confidence intervals based on 1,000 resamples) shed light on the pattern of this interaction. In essence, this pattern mainly supports Hypothesis 2. Male and female leaders believed the follower to be a good successor when there was either fit or in the control condition where they merely received competence information. Importantly, however, when they received information that there was *lack* of fit, male leaders rated the followers’ successor potential significantly lower (*M* = 4.71) than female leaders (*M* = 5.21; *p* = 0.004). Accordingly, male leaders’ ratings of successor potential were significantly reduced when there was lack of fit than in the neutral (*M* = 5.47, *p* < 0.001) or fit conditions (*M* = 5.42, *p* < 0.001). In contrast, the female leaders’ successor evaluation was less dependent on fit, such that the lack of fit condition outcomes only differed marginally from the fit conditions, *M* = 5.53, *p* = 0.07) and not from the control condition. A visual representation of the interaction pattern is provided in Figure 4.

Notably, when we did include the percentage of female leaders currently working in leaders’ organizations as a control variable in this analysis, the interaction term of the 2 by 3 ANOVA on followers’ successor potential dropped to non-significance, *F*(2, 302) = 2.04, *p* = 0.13, *η* = 0.014. However, the simple main effects within the no-fit condition remains significantly different for male and female leaders (*p* = 0.019).

In addition, we also explored in a supplementary analysis whether the interactive effect of leader gender and interpersonal fit on followers’ successor potential could be explained by the power levels of male and female leaders. To examine this possibility, we ran the mediated (second stage) moderation Model 14 of Hayes (2013), 95% *CI*, 5000 bootstraps, which first tested whether leader gender predicted differences in leader power, before testing whether these power differences, depending on the interpersonal fit information leaders received, significantly predicted the different successor evaluations. The results showed that leader power significantly predicted followers’ successor potential (*B* = 0.26, *t* = 2.02, *p* = 0.04) and that the differential power perceptions of male and female leaders were a significant driver of their successorship evaluations across all three conditions (neutral: *CI*: −0.15 to −0.01, fit: *CI*: −0.13 to −0.01, lack of fit; *CI*: −0.13 to −0.003). This means that the gendered power differences and the fit manipulation did not jointly predict their evaluations.

### Discussion

In general, the findings of this experimental study largely confirm our second hypothesis that leader gender would moderates the relationship between interpersonal fit and followers’ successorship potential. We indeed found that this relationship is significantly stronger for male leaders than for female leaders. However, it was not the presence of interpersonal fit that affected male leaders’ successor potential ratings. Rather, it was the *absence* of fit that caused male leaders to respond negatively to potential successors. As anticipated, however, interpersonal fit perceptions did not inform female leaders’ ratings of followers’ successor potential. Indeed, presumably because of the high competence level of the follower, female leaders rated the potential successor equally positively across all three conditions. Notably, the inclusion of a neutral baseline condition allowed us to discover that without further information, all leaders assume relatively high levels of interpersonal fit between themselves and their followers.

In conclusion, this study provides further evidence for our central proposition that there are gender differences in how leaders rely on interpersonal fit to determine succession potential. Interestingly, in supplementary analyses, we also found that the female leaders in our sample felt they had relatively little power in their organization compared to the male leaders, in spite of formally holding similar management positions. We will elaborate on this additional finding in section “General Discussion.”

## General Discussion

The core research idea we aimed to prove in this contribution is that the relative importance of interpersonal fit when leaders evaluate followers for their potential as successors is likely to be gendered. Our central proposition was that male leaders would be more likely to select interpersonally similar followers as potential successors than female leaders.

## Summary of Research Findings

We conducted a multisource field study and a vignette study among male and female leaders to examine this proposition. In Study 1, we tested our first hypothesis that the relationship between leader gender and their ratings of followers’ successor potential would hinge on follower’s evaluations of positive leader behaviors that tend to signal close relations and interpersonal fit (i.e., evaluations of leaders being coaching, leading by example, and transformational, see Hypothesis 1). The results confirm that for male leaders, positive follower evaluations on the three leadership styles associated significantly with their ratings of followers’ successor potential. By contrast, for female leaders, positive follower evaluations of the leadership styles were unrelated or even significantly negatively related to their ratings of followers’ successor potential. Hence, Study 1 offers initial evidence that male leaders are more likely to take interpersonal fit perceptions into account when making successor judgments than female leaders. We conducted a second experimental study to examine the role of interpersonal fit in successor decisions more directly. Replicating Study 1, we again found that male leaders, compared to female leaders, attach greater importance to interpersonal fit perceptions in their successor ratings.

## Theoretical Relevance of Findings

Current theorizing on the root causes underlying gender inequality in the work place suggests that the emphasis that male leaders’ place on social similarities and fit in selecting their prospective successors represents a key contributor to the underrepresentation of women in leadership positions (Byrne, 1971; Koch et al., 2015). Empirically, however, there is ambiguity about the existence of leader gender differences in the reliance on fit perceptions in developing informal networks (McDonald, 2011). Prior observed effects are obtained primarily through archival data and could thus be explained by women’s lack of opportunities in creating the social support they desire, rather than by their preferences for fit (Fischer and Oliker, 1983; Moore, 1990). Moreover, with such data, it is impossible to establish whether psychological interpersonal fit indeed explains why (male) management networks are homogeneous in nature. We therefore believe that our systematic comparison of male and female leaders’ responses to interpersonal fit when informally selecting successors for future leader roles has important theoretical implications for gender research. First, the results across the two studies largely confirm our central proposition that male leaders consider interpersonal fit more relevant in their evaluations of potential successors, hereby supporting the idea that male leaders’ informal network choices (perhaps unintentionally) keep the old boys’ network in place. As past research has demonstrated that such networks generate great benefits for the career advancement of male followers (Garman and Glawe, 2004; Linehan and Scullion, 2008), our findings indeed imply that male leaders’ preference for interpersonal fit may inadvertently create barriers to women’s career progression (McDonald, 2011).

Second, our finding that female leaders, compared to their male counterparts, give less weight to fit perceptions when evaluating potential successors has important implications for the ongoing debate on women’s successor and sponsorship strategies once in power. Our finding suggests that women are neither “change agents,” as they seem not to prefer similar others, such as other women, nor are they simply “cogs in a machine,” as they do not tend to prefer dissimilar others either (i.e., men; Lyness and Thompson, 2000; Cohen and Huffman, 2007; Stainback et al., 2016). Rather, female leaders tend to disregard issues of interpersonal fit when making succession judgments. One may argue that female leaders are fairer when it comes to succession decisions, being less swayed by interpersonal fit and instead relying simply on the competence of the potential successor. Hence, our work points out that in attempting to erode gender inequality, we need to not only alleviate selection biases that prevent women from entering leadership positions in the first place but also we need to better understand women’s perceptions of followers once they are in a leadership position.

## Limitations and Future Research Directions

The current research is not without limitations. Study 1 provided evidence that the relationship between leader gender and successorship potential depended on interpersonal fit perceptions as signaled by followers’ evaluations of leadership styles (i.e., coaching, leading by example, and transformational leadership). Nevertheless, it is possible that these leadership styles reflect perceptions other than interpersonal fit that could explain male leaders’ successor ratings. That is, although underlying social similarities are generally assumed to guide positive leader evaluations (Kanter, 1977), we cannot give full certainty that this is the case in our data. Consequently, there may be alternative explanations for our Study 1 findings. To give one concrete example, transformational leadership is known to be a visionary leadership style that motivates followers to look beyond their current abilities and position (Bass and Avolio, 1990). The male leaders who received high scores on this style could thus also consider career advancement important and were therefore more attuned to their followers’ successor potential. This alternative reasoning cannot explain, however, why positive evaluations of female leaders’ on these styles were unrelated to their follower successor ratings. Nonetheless, with this in mind, we would encourage future field research to re-examine which leadership style is most likely to create perceptions of leader-follower fit, use both leader and follower ratings to objectively assess the amount of fit in these perceptions, and then test whether these fit perceptions matters for the career progression of followers.

To alleviate some of Study 1’s limitations, in Study 2, we did measure interpersonal fit more directly (and independently of followers’ levels of competence to fulfill a future leader position). This leadership study also had the benefit of ensuring high internal validity and addressing issues of causality. Still, two points remain open for discussion. First, our operationalization of, and findings on, interpersonal fit follows directly from Byrne’s (1971) fit definition as representing positive interpersonal relationships guided by social similarities that facilitates resources exchanges. However, we did not find any (moderating) effects for leader-follower gender fit in both studies. On the one hand, this finding underlines that fit perceptions can be derived from multiple sources of demographic and social similarities (Byrne, 1971). On the other hand, it may be that in our samples, leaders were unresponsive to gender fit due to social desirability concerns (Richman et al., 1999). In this regard, if we want to fully understand the old boys’ network phenomenon and the ways to overcome its existence, more research is needed on how leader-follower gender fit and interpersonal fit perceptions are exactly related to each other.

Second, the supplementary analyses we conducted in Study 2 revealed that female leaders reportedly held less power than male leaders. This result resonates with the idea that female leaders often feel socially isolated at the top and believe that they not adequately represent the behaviors typically expected from (male) leaders in their organization (Heilman, 2001; Derks et al., 2016). Importantly, these differential power perceptions of male and female leaders also had an impact on followers’ successor evaluations. We therefore further tested whether female leaders considered it less relevant, or appropriate even, to evaluate the follower’s successor potential based on interpersonal fit *because* of their lowered power perceptions. However, the link between leaders’ perceived power and their successorship ratings did not hinge on the presence of leader-follower fit. This means that the little emphasis female leaders’ place on interpersonal fit in evaluating successor potential cannot be explained by their perceptions of not being a typical organizational manager themselves. Accordingly, it remains open for further investigation what other key mechanisms could potentially explain why female leaders are less attuned to fit perceptions than are male leaders.

One possible alternative explanation of our findings could be that female leaders may be less likely to show selection biases and rely less on interpersonal fit perceptions because they are more aware of discriminatory practices than male leaders are (Maume, 2011). This possibility underscores Ibarra’s (1997) theoretical reasoning that female leaders’ sponsorship strategies are different from those of male leaders because female leaders are more concerned with being competent and demonstrating their worth to the organization than are male leaders. We therefore believe that future research in this area should include indicators of the degree to which female leaders attempt to legitimizing their own position within the organization and test whether these attempts can explain why male and female leaders differ in their focus on interpersonal fit in promoting followers.

Finally, more generally, our focus on fit as a psychological mechanism underlying the old boys’ network phenomenon highlights the importance of examining the unique strategies male leaders use to sponsor their follower into successor roles. In this regard, we support the recent call of scholars to not only focus on the obstacles that women and members of other underrepresented groups face as they try to succeed professionally. Future research should also include the experiences of men, as they are equally important for understanding why workplace gender discrimination is so persistent (Bruckmüller et al., 2013).

## Conclusion and Practical Implications

For companies seeking a clear road map for supporting and advancing women’s position in the work place, we advise them to not only make a compelling case for gender diversity, or to ensure that formal hiring, promotions, and reviews are fair. First, given that organizational success stems from “capturing the value of the entire workforce, not just a few superstars” (O’Reilly and Pfeffer, 2000, p. 52, see also Steffens et al., 2018), less reliance on the individual sponsor activities of leaders and a greater focus on a more systematic, inclusive HR approach is recommended. With such an inclusive approach, organizations offer equal developmental and network opportunities to all employees at lower management position (Gallardo-Gallardo et al., 2013). Second, if organizations do rely on leader perceptions of successor potential, they need to ensure that upper management is held more accountable for their sponsorship activities and the resulting career advancements that they provide to specific followers (and not others).

## Ethics Statement

This study was carried out in accordance with the recommendations of “Behavioral Research Lab’s ethical committee of the Faculty of Economics and Business, University of Groningen, The Netherlands (Committee president: Dr. Marijke Leliveld)” with written informed consent from all subjects. All subjects gave written informed consent in accordance with the Declaration of Helsinki. The protocol was approved by the “Behavioral Research Lab’s ethical committee of the Faculty of Economics and Business, University of Groningen, The Netherlands.”

## Author Contributions

Each author has made substantial contributions to the conception or design of the work. FR, JS, MR, and NS jointly drafted the content of the paper, where FR was in the lead. AN, JS and FR collected, analyzed, and interpreted the data of Study 1. JS and FR collected, analyzed, and interpreted the data of Study 2. All authors provide approval for publication of the content and agree to be accountable for all aspects of the work in ensuring that questions related to the accuracy or integrity of any part of the work are appropriately investigated and resolved.

### Conflict of Interest Statement

The authors declare that the research was conducted in the absence of any commercial or financial relationships that could be construed as a potential conflict of interest.
