# Supplementary material for: Gender Differences in How Leaders Determine Succession Potential: The Role of Interpersonal Fit With Followers
Source: Front Psychol. 2019 May 3;10:752. doi: 10.3389/fpsyg.2019.00752 (PMC6509144; doi:10.3389/fpsyg.2019.00752)
Supplement: Supplementary file 1 [file Data_Sheet_1.docx]

*Suppementary Figure 1.* Interactive relationship of followers’ positive leader evaluation and leader gender with leaders’ assessment of followers’ successorship potential (Study 1).
